# Level of depression, anxiety and stress in patients with intrauterine adhesions in Hunan Province, China: A cross-sectional study

**DOI:** 10.1371/journal.pone.0229832

**Published:** 2020-03-11

**Authors:** Ting Wang, Yanhui Zhou, Jingxia Fu, Mingzhu Chen, Yang Luo

**Affiliations:** 1 Departmnt of Nursing, The People’s Hospital of Longhua, Shenzhen, China; 2 Department of Obstetrics and Gynecology, Xiangya Nursing School of Central South University, Changsha, Hunan, China; USC Keck School of Medicine, Institute for Global Health, UNITED STATES

## Abstract

**Background:**

The incidence of intrauterine adhesions has been increasing in recent years, seriously affecting women’s health. This study aimed to investigate the psychological status and identify risk factors associated with high psychological distress in patients with intrauterine adhesions.

**Methods:**

A cross-sectional study was conducted in Hunan Province, China. A total of 258 patients who presented with intrauterine adhesions between February and May 2018 were included. Data were collected by a questionnaire packet that included the Depression Anxiety Stress Scale, the Medical Coping Mode Questionnaire, and demographic and clinical information. Descriptive statistics, t-tests, one-way ANOVA, Pearson’s correlations and multiple linear stepwise regression were employed in this study.

**Results:**

Among 258 participants, the detection rates of mild depression and moderate to extremely severe depression were 10.1% and 10.5%, respectively; the detection rates of mild anxiety and moderate to extremely severe anxiety were 11.2% and 20.2%, respectively; the detection rates of mild stress and moderate to extremely severe stress were 9.3% and 10.2%, respectively. Avoidance and resignation coping were positively correlated with the overall scores of general distress which represents the total scores of the Depression Anxiety Stress Scale (r = 0.171, 0.475, P < 0.01). Multiple linear stepwise regression results showed that husband-wife relationships and avoidance and resignation coping strategies were the main factors predicting general distress levels.

**Conclusions:**

Patients with intrauterine adhesions have psychological distress in a certain extent. Target interventions should be taken to improve the mental health level of patients.

## Introduction

An intrauterine adhesion (IUA) is a recognized cause of secondary amenorrhea and, consequently, secondary infertility [1, 2]. It is a form of disease that causes the uterine muscle walls to adhere to each other due to the basal layers of the endometrium being damaged by various factors; thus a partial or complete occlusion of the uterine cavity, also known as Asherman syndrome[3]. According to statistics, the incidence of infertility caused by IUA is 43%, and the incidence of IUA in infertility patients is as high as 13% [4]. The incidence of IUA caused by uterine cavity operation is 25%-30%, and the incidence of IUA caused by curettage after abortion is 93% [5, 6]. At present, with the progress of medicine, transcervical resection of adhesions is the best treatment for IUA, but the recurrence of IUA and infertility after an operation are still problems to be solved [7]. In recent years, with the increase in uterine cavity surgery, IUA incidence has been increasing, seriously affecting women’s reproductive function, menstrual physiology, and physical and mental health [8, 9].

At present, China has joined the ranks of “low fertility rate countries.” The universal two-child policy that allowed all couples to have two children was approved and formally implemented on January 1, 2016. With the introduction of the two-child policy and the urgent desire of patients for fertility, IUA as an important cause of secondary infertility has attracted increasing public attention in China. Childbearing is considered as a family obligation in Chinese culture [10]. Women will experience a variety of negative emotions and bear tremendous psychological pressure when reproductive dysfunction occurs. A previous study indicated that the overall rate of re-adhesion after operation for intrauterine adhesions is 3.1%~23.5% and the rate of severe adhesions is as high as 62.5% [7]. Because of its long treatment cycle and readiness to recur, patients are more likely to occur self-blame and have negative emotions. Yang Huan et al [11] found that the incidence of depression and anxiety in 101 patients with intrauterine adhesions was 59.4% and 24.8%, respectively, which was significantly higher than that in the general population. Researches also demonstrated that long-term psychological distress not only seriously affects the quality of life and health of infertile patients but also may affect their fertility outcome [12, 13].Therefore, it is of great significance to investigate current psychological well-being and related factors among patients with IUA for improving their overall health.

To the best of our knowledge, current studies on IUA patients have focused on aetiology, diagnosis and treatment. There is a lack of understanding of the psychological status and related factors concentrated on patients with IUA. This study, therefore, examined the emotional distress of IUA patients and identified specific factors associated with higher emotional distress.

## Methods

### Design and sample

A cross-sectional study was conducted in one of the largest university-affiliated hospitals actively treating IUA patients in Hunan, China, to achieve representative data. Participants were included if they were married, aged between 18 and 49 years, diagnosed with IUA and were treated by hysteroscopy as inpatients in the Departments of Obstetrics and Gynaecology. Participants were excluded if: (a) they had chronic diseases such as diabetes, heart disease, or hypertension; or (b) they were unable to understand and complete the questionnaires or provide oral informed content; or (c) they were with history of chronic depression or anxiety or who that used medication for this problems. Data were collected between February and May 2018. This study was approved by the Ethical Committee of Central South University on 24 December 2017. The approval number is 2017026. Verbal informed consent was obtained from all participants.

### Measures

#### Demographic and clinical information

The study inquired about the age, residence, educational level, personal monthly income, family annual income, self-perceived husband-wife relationship (measured by the question “How do you think of your relationship with your husband”, with three answers: “Less Harmonious”, “Harmonious”, and “Very harmonious”), treatment purpose, number of children, number of admissions which related to the recurrence of the IUA, awareness of disease, and disease severity.

#### Depression Anxiety Stress Scale (DASS21)

The DASS21 is a set of three self-report scales that contains 7 items per scale designed to measure the negative emotional states of depression, anxiety and stress [14]. Respondents are required to indicate the presence of these symptom(s) over the past week on a four-point Likert scale ranging from 0 (“did not apply to me at all”) to 3 (“applied to me very much”). High scores suggest that the symptomatology is severe. The recommended cut-offs points for each subscale are as follows: depression [normal (0–9), mild (10–13), moderate (14–20), severe (21–27), and extremely severe (28+)], anxiety [normal (0–7), mild (8–9), moderate (10–14), severe (15–19), and extremely severe (20+)], and stress [normal (0–14), mild (15–18), moderate (19–25), severe (26–33), and extremely severe (34+)][14, 15]. The DASS21 was translated into Chinese and reported satisfactory psychometric properties [16]. In this study, the Cronbach’s alpha for the depression, anxiety and stress subscales were 0.823, 0.819 and 0.818, respectively. This study also used the overall score (ranging from 0 to 126) to indicate general distress.

#### Medical Coping Mode Questionnaire (MCMQ)

The MCMQ contains 20 items covering three subscales, namely, confrontation, avoidance, and resignation, which reflect the basic reactions of people at risk [17]. The Chinese version was revised, analysed and tested by Qianjin Jiang and Xiaohong Shen. In the Chinese version, the Cronbach’s alpha coefficient of the three dimensions was found to be 0.69, 0.60 and 0.76, respectively, and the test-retest reliability was 0.64, 0.85, and 0.67, respectively. The MCMQ has been found to have satisfactory psychometric properties [18]. The Cronbach’s alpha for the three dimensions was 0.60, 0.76 and 0.69, respectively, in this study.

### Statistical analyses

A database was created by EpiData 3.0. All statistical analyses were performed using SPSS 23.0 (SPSS Inc., Chicago, IL) software. Categorical data were described with percentages and numbers, and continuous data were described with means and SD. Continuous variables were analysed with t-tests and one-way ANOVA. Pearson’s correlations were used to test for associations between variables. Descriptive statistics summarized the socio-demographic characteristics of the participants as well as the clinical characteristics and level of depression/anxiety/stress. Differences in the scores of total DASS21 among groups with different socio-demographic and clinical characteristics were computed using a t-test or one-way ANOVA. The relationship between coping strategies and psychological status (depression, anxiety, stress and total DASS21) was explored using Pearson’s correlations. Variables identified to be significant (p<0.05) in these initial tests were then entered in a multiple linear stepwise regression model to determine predictors of higher general distress.

All tests were two-tailed, with alpha set at p < 0.05.

## Results

### Socio-demographic characteristics

A total of 258 eligible IUA patients were interviewed in this study. The mean age of the participants was 32.0 years (SD 5.3; range 21–48 years). The purpose of treatment was fertility for most (93.8%) participants in this study. More than half (52.3%) had at least junior college education and had no children (55.8%). The majority (66.7%) were urban residents and had personal monthly incomes over 847.4 $ (57.0%). Nearly half of the participants had an annual household income over 14123.3 $ (43.8%). More than half (57.0%) had a very harmonious husband-wife relationship. Approximately one-third (33.0%) had better awareness of the disease. Most participants (85.7%) had moderate to severe IUA, and 33.9% had at least three hospitalizations due to intrauterine adhesions. (Table 1)

**Table 1:** Demographic and clinical information and associations between demographic/clinical factors and general distress score (n = 258).

	N (%)	General distress score	t/F value	*P* value
Age (years)			1.31	0.192
≤30	117(45.3%)	22.7±18.4		
>30	141(54.7%)	20.0±14.6		
Level of education			3.57*	0.015
Junior high school	54(20.9%)	23.6±15.0		
Middle special/senior high school	69(26.7%)	24.2±15.5		
Junior college	81(31.4%)	21.2±19.7		
College graduate or higher	54(20.9%)	15.3±11.9		
Residence			4.01**	0.000
Rural area	86(33.3%)	27.4±19.2		
Urban area	172(66.7%)	18.1±14.0		
Family annual income ($)			8.17**	0.000
< 7061.6	56(21.7%)	30.3±19.9		
7061.6–14123.2	89(34.5%)	19.3±14.5		
14123.3–21184.8	55(21.3%)	19.6±16.1		
>21184.9	58(22.5%)	16.9±12.6		
Personal monthly income ($)			2.16*	0.031
≤847.4	111((43.0%)	23.7±17.9		
>847.4	147(57.0%)	19.3±15.1		
Number of children			1.58	0.116
0	144(55.8%)	22.7±16.9		
≥1	114(44.2%)	19.4±15.7		
Husband-wife relationship			15.78**	0.000
Less Harmonious	47 (18.2%)	31.7±19.5		
Harmonious	100(38.8%)	21.6±15.7		
Very harmonious	111(43.0%)	16.5±13.5		
Purpose of treatment			-0.748	0.455
Fertility	242 (93.8%)	21.43±16.50		
Recovery of the menstruation	16 (6.2%)	18.25±15.91		
Severity of IUA			-3.20**	0.002
Mild	37(14.3%)	15.4±11.0		
Moderate to severe	221(85.7%)	22.2±17.0		
Number of adimission			2.45	0.064
1	81(31.4%)	23.5±1.9		
2	87(33.7%)	19.4±1.7		
3	55(21.3%)	18.1±1.9		
≥4	35 (13.6%)	25.7±3.4		
Awareness of IUA			2.71*	0.031
Very well known	27(10.5%)	20.4±18.8		
Better known	58(22.5%)	18.2±13.3		
Known	107(41.5%)	19.9±16.4		
A little known	55(21.3%)	25.2±17.2		
Don’t know	11(4.2%)	32.0±18.0		

*statistically significant by one-way ANOVA or t test with P < 0.05

**statistically significant by one-way ANOVA or t test with P < 0.0l

### Level of psychological status

The total DASS21 score of the participants was 21.23 (SD 16.5), the anxiety score was 5.88 (SD 5.5), the depression score was 5.49 (SD 5.5), and the stress score was 9.86 (SD 7.1). The detection rates of mild depression and moderate to extremely severe depression in this study were 10.1% and 10.5%, respectively. The detection rates of mild anxiety and moderate to extremely severe anxiety were 11.2% and 20.2%, respectively. The detection rate of mild stress was 9.3% and that of moderate to extremely severe stress was 10.2%.

### Correlations between coping strategies and psychological status

Intercorrelations between coping strategies and measures of psychological status (depression, anxiety, stress and total DASS21) were explored (Table 2). Most of the correlations were significant except for confrontation. The findings suggested that measures of psychological status were positively and significantly related to avoidance and resignation.

**Table 2:** Correlation between psychological measures and medical coping mode among participants.

Medical coping mood	DASS21-Depression	DASS21-Anxiety	DASS21-Stress	DASS21-General distress
Confrontation	0.058	0.030	0.035	0.045
Avoidance	0.136*	0.143*	0.178**	0.171**
Resignation	0.448**	0.389**	0.449**	0.475**

*statistically significant by Pearson’s correlations with P < 0.05

**statistically significant by Pearson’s correlations with P < 0.0l

### Predictors of higher general distress

According to the results of t-tests and one-way ANOVA, patients were likely to obtain higher scores of general distress if they were rural residents (t = 4.01, p<0.01), had low education levels (F = 3.57, p<0.05), low personal monthly income (t = 2.11, p<0.05), low family annual income (F = 8.17, p<0.01), a poor relationship between husband and wife (F = 15.78, p<0.01), less awareness of the IUA disease (F = 2.71, p<0.05) or a severe case of the disease (t = -3.21, p<0.01) (Table 1).

Variables that had a significant correlation with higher DASS21 scores were retained in a multiple linear stepwise regression model. A best-fit regression indicated that the husband-wife relationship, avoidance, and resignation coping strategies were all significant predictors of higher general distress among our participants (Table 3, Table 4).

**Table 3:** Independent variable assignment.

Independent variable	Assignment
Residence	Rural area = 1, Urban area = 2
Level of education	Junior high school = 1, Middle special/senior high school = 2, Junior college = 3, College graduate or higher = 4
Personal monthly income	≤847.4 $ = 1, >847.4 $ = 2
Family annual income	<7061.6 $ = 1, 7061.6–14123.2 $ = 2, 14123.3–21184.8 $ = 3, >21184.9 $ = 4
Husband-wife relationship	Less Harmonious = 1, Harmonious = 2, Very harmonious = 3
Severity of IUA	Mild = 1, Moderate to severe = 2
Awareness of IUA	Very well known = 1, Better known = 2, Known = 3, Known a little = 4, Don’t know = 5

**Table 4:** Multivariate linear stepwise regression analysis of general distress among participants (n = 258).

Predictors	B	SE	Beta	*t* value	*P* value
Husband-wife relationship	-5.382	1.295	-0.245	-4.158	0.000
Resignation coping	2.665	0.382	0.411	6.979	0.000
Avoidance coping	0.965	0.351	0.159	2.746	0.007

B unstandardized coefficients, SE standard error, Beta (β’) standardized coefficients

*R*^*2*^ = 0.299, adjusted *R*^*2*^ = 0.289, *F* = 30.034, *P<*0.001

## Discussion

This is the first study to examine the current psychological well-being and factors that influence the tendency towards high levels of depression, anxiety and stress among patients with IUA. With the increasing incidence of IUA, the opening of the second-child policy and the urgent desire of patients for fertility, IUA patients have attracted increasing attention in contemporary China. Our study revealed that the prevalence of anxiety and stress in IUA patients were higher than that in patients with Rheumatoid Arthritis using the same measurement[19]. Different characteristics of the subjects may be the cause of these differences. In addition, in comparison to some of the unselected/control populations in previous studies using the same scale—DASS21, we found that the total DASS score in our study do appear to be elevated which can partly proved that IUA patients had a relatively high level of psychological distress [20–23]. Furthermore, patients who had a history of chronic anxiety or depression (or prior medication for either) had been excluded, which means that levels of anxiety and depression in unselected populations with IUA may be even greater than those reported in our study. In this study, majority (93.8%) participants had strong fertility desires. In Chinese communities, the culture’s emphasis on honouring the family through procreation has contributed to the plight of infertile couples [10]; thus, they are more likely to suffer from anxiety, depression, stress and other psychological distress. Therefore, understanding current psychological well-being and factors related to psychological disorders among patients with IUA will help to develop targeted interventions to promote the mental health of our patients.

Level of education, awareness of IUA, residence, personal monthly income, family annual income and disease severity were all shown to be significantly correlated with higher levels of general distress in the present study. Studies have shown that patients with better educational backgrounds may experience lower levels of depressive symptoms among infertile women, which is consistent with our study [24]. Better education may help patients have more access to health information, which can partly account for these results. However, a study in Iran found no association between educational level and mental health status [25]. In addition, awareness of IUA was a significant variable in the ANOVA output, which suggested that reliable disease information is needed for patients; however, it was not significant in the multiple regression model. A systematic review showed that patients with fertility problems were more likely to have decreased infertility-related stress after counselling intervention [26]. Therefore, health professionals should pay significant attention to providing patients with information relating to disease and treatment procedures to reduce negative emotions in the course of treatment.

The IUA patients were more likely to experience general distress with low economic status. The long treatment cycle and lack of medical insurance coverage for IUA increase the family’s economic burden. Researchers claimed that depression decreased as the economic status increased [27]. Incorporating IUA treatment into medical insurance coverage may lessen the financial burden for patients. In addition, the results of this study suggested that patients who lived in rural areas were more likely to suffer mental problems. Cultures emphasize the critical role of reproduction in rural areas. These findings suggest that targeted measures should be taken to resolve emotional distress, especially for rural women [28].

The present study also found that a negative husband-wife relationship could predict higher level of general distress, which confirmed that a harmonious husband-wife relationship can effectively alleviate the psychological pressure of patients in the course of treatment. A previous study by Matsubayashi also showed that childless Japanese women lacking husband support had high levels of emotional distress, anxiety, and depression [29]. Another study also indicated that women with good communication with partners were less likely to experience distress when facing infertility [30]. On the other hand, it is well documented that higher levels of stress and negative mood might impair the marital relationship in turn which may lead to an unfavorable cycle [31, 32]. As bearing children is often considered necessary to fulfil marital roles, couples who cannot do so may feel the essence of their relationship is threatened. The diagnosis of infertility can engender shame, guilt, anger, sadness, and loss of control in both women and their husbands [33]. Thus, requiring the participation and cooperation of partners in the course of treatment and psychological interventions targeted for both wife and husband may help to promote the mental health of patients [34].

Furthermore, the findings of the present study also highlighted that avoidance and resignation coping strategies were positively related to high levels of depression, anxiety and stress. These findings were similar to previous studies in other populations [35, 36]. A study conducted in Iran found that the majority of infertile women choose to avoid the psychological stress caused by infertility and avoid social activities with family and friends, which means that they were more inclined to adopt maladjustment mechanisms [37]. Previous studies have found that coping strategies should be regarded as an important factor in determining the overall health status of patients. Coping strategies can indirectly change the physiological and psychological status of patients and affect the treatment efficacy and quality of life of patients [38]. Sufficient comprehensive care management programs, including psychological counselling, cognitive reconstruction, and strengthening the social support system, were found to effectively reduce the negative coping mood (avoidance and resignation) and increase the confrontation coping strategy, which helped infertile patients overcome their negative emotions and become confident they could overcome the disease[39]. Another study also indicated that a group-based cognitive behavior stress management program not only reduced anxiety and depression levels but also reduced negative coping and increased social support [40]. It is therefore important to focus on the characteristics of coping styles of patients and formulate reasonable intervention measures to enable patients to effectively cope with and reduce the psychological burden of the disease.

In addition, it is worth noting that the total DASS21 score showed no differences between number of admissions in this study, but the score had a U-shaped distribution as the number of admissions increased. The patients who were at first admission or at more than three admissions were more likely to experience a high level of psychological distress than patients who had two to three admissions. The possible reasons may be that patients are more prone to experience various psychological problems because of an unknown environment, treatment methods or personal physical reactions for the first treatment, while in the process of continuing treatment, the general distress level gradually decreases due to continuous adaptation. However, when the disease has not improved or the treatment effect is poor and further treatment is still needed, the distress of patients increases sharply, and the psychological burden increases [26]. Therefore, it is very important to pay attention to the treatment process of individual patients and to take psychological intervention measures at the right time.

Several limitations of this study should be noted. The first limitation concerns the measures used: our definition of “psychological distress” was based on a self-rated questionnaire and not on a clinical diagnostic interview. Second, the current study was carried out in only one inpatient hospital ward of the Gynaecology Department in Changsha; thus, our findings may not be generalizable to other populations. In the future, researchers should perform multicentre studies. Third, the study didn’t inquire the type of previous deliveries which is an important question. Further studies should add such factors to make in-depth analysis. However this study focus on the IUA related clinical information including the number of admissions related to the recurrence and disease severity; thus, this study could make significant contributions for IUA patients to some degree. In addition, due to the limitation of the study design without a control group, we failed to make a definite conclusion whether the level of depression, anxiety and stress in IUA patients was high or low. Despite these limitations, this study is the first to determine the prevalence of and demographic/clinical/coping strategies associated with general distress and to innovatively introduce the variables of marital relationship and coping style among IUA patients affected by psychological distress. In addition, this study may provide insight into the intervention targeted for IUA which have important implications on clinical practice and future research.

## Conclusion

In conclusion, patients with IUA have psychological distress in a certain extent. Higher distress was significantly, negatively associated with the husband-wife relationship and positively, significantly associated with avoidance and resignation coping behaviours in IUA patients. Target interventions are needed to reduce general distress in IUA patients. Husbands might be key figures in performing general distress-reducing interventions to maintain and enhance mental health among IUA patients. Psychological counselling, better access to disease information and other support may be needed to improve effective coping in such interventions.
